# The Angiotensin-(1-7)/Mas Axis Counteracts Angiotensin II-Dependent and -Independent Pro-inflammatory Signaling in Human Vascular Smooth Muscle Cells

**DOI:** 10.3389/fphar.2016.00482

**Published:** 2016-12-15

**Authors:** Laura A. Villalobos, Álvaro San Hipólito-Luengo, Mariella Ramos-González, Elena Cercas, Susana Vallejo, Alejandra Romero, Tania Romacho, Raffaele Carraro, Carlos F. Sánchez-Ferrer, Concepción Peiró

**Affiliations:** ^1^Department of Pharmacology, School of Medicine, Universidad Autónoma de MadridMadrid, Spain; ^2^Service of Endocrinology, Hospital de La PrincesaMadrid, Spain; ^3^Department of Medicine, School of Medicine, Universidad Autónoma de MadridMadrid, Spain

**Keywords:** vascular smooth muscle, inflammation, inducible nitric oxide synthase, angiotensin-(1-7), Mas receptors, cytokines, interleukin-1β, cell signaling

## Abstract

**Background and Aims:** Targeting inflammation is nowadays considered as a challenging pharmacological strategy to prevent or delay the development of vascular diseases. Angiotensin-(1-7) is a member of the renin-angiotensin system (RAS) that binds Mas receptors and has gained growing attention in the last years as a regulator of vascular homeostasis. Here, we explored the capacity of Ang-(1-7) to counteract human aortic smooth muscle cell (HASMC) inflammation triggered by RAS-dependent and -independent stimuli, such as Ang II or interleukin (IL)-1β.

**Methods and Results:** In cultured HASMC, the expression of inducible nitric oxide synthase (iNOS) and the release of nitric oxide were stimulated by both Ang II and IL-1β, as determined by Western blot and indirect immunofluorescence or the Griess method, respectively. iNOS induction was inhibited by Ang-(1-7) in a concentration-dependent manner. This effect was equally blocked by two different Mas receptor antagonists, A779 and D-Pro^7^-Ang-(1-7), suggesting the participation of a unique Mas receptor subtype. Using pharmacological inhibitors, the induction of iNOS was proven to rely on the consecutive upstream activation of NADPH oxidase and nuclear factor (NF)-κB. Indeed, Ang-(1-7) markedly inhibited the activation of the NADPH oxidase and subsequently of NF-κB, as determined by lucigenin-derived chemiluminescence and electromobility shift assay, respectively.

**Conclusion:** Ang-(1-7) can act as a counter-regulator of the inflammation of vascular smooth muscle cells triggered by Ang II, but also by other stimuli beyond the RAS. Activating or mimicking the Ang-(1-7)/Mas axis may represent a pharmacological opportunity to attenuate the pro-inflammatory environment that promotes and sustains the development of vascular diseases.

## Introduction

Vascular inflammation is a key feature of vascular diseases, like hypertension or atherosclerosis, which are major causes of mortality worldwide (Daiber et al., 2016). In the context of vascular diseases, vasoactive peptides, cytokines, and other agents, play a pivotal role in promoting vascular inflammation not only through the early recruitment of circulating leukocytes, but also during the progression of the disease through the activation of vascular smooth muscle cells (VSMC) (Lacolley et al., 2012; Chistiakov et al., 2015). Indeed, activated VSMC can switch to a pro-inflammatory phenotype that drives the expression of a wide array of pro-inflammatory molecules and mediators, which in turn perpetuate and amplify the deleterious vascular environment (Lacolley et al., 2012; Chistiakov et al., 2015). In this context, attenuating inflammation, by pharmacological tools or other approaches, is nowadays considered as a challenging strategy to prevent or delay the development of vascular diseases (Everett et al., 2013; Daiber et al., 2016).

The renin-angiotensin system (RAS) is a key player in the regulation of cardiovascular homeostasis both in health and disease. Angiotensin (Ang)-II has been extensively studied over the last decades in the context of vascular pathophysiology, where it has proven to play a pivotal role in disrupting vascular homeostasis and promoting endothelial dysfunction and vascular remodeling through the activation of AT1 receptors (Balakumar and Jagadeesh, 2014). Ang II has also been extensively linked to vascular cell inflammation, through signaling pathways involving the generation of reactive oxygen species (ROS) and the activation of the transcription factor nuclear factor (NF)-κB, among other (Montezano et al., 2014).

While Ang II has been for long acknowledged as the main biologically active peptide of the RAS, other components of the RAS have recently gained growing attention. Ang-(1-7) is a heptapeptide, mainly generated from Ang II through the action of angiotensin converting enzyme 2 (ACE2) but also from Ang I via neutral endopeptidase (NEP) activity (Passos-Silva et al., 2015). Ang-(1-7) is a ligand for the G protein-coupled receptor Mas (Kostenis et al., 2005). In the vasculature, Ang-(1-7) has been considered as a physiological antagonist of Ang II, since it displays vasodilatory, antiangiogenic, antiproliferative, antimigratory, antiapoptotic, and antihypertrophic properties, among other (Jiang et al., 2014; Passos-Silva et al., 2015). Several of these vasculoprotective actions are mainly the result of the modulation of VSMC function by Ang-(1-7) (Zhang et al., 2010; Alzayadneh and Chappell, 2014; Bihl et al., 2015). Little is known, however, on the direct anti-inflammatory properties of the heptapeptide on this vascular cell type.

In the present study, we evaluated the capacity of Ang-(1-7) to counteract the pro-inflammatory response triggered by Ang II in primary human VSMC cultures in terms of NF-κB activation and inducible nitric oxide synthase (iNOS) induction. Moreover, we tested the capacity of Ang-(1-7) to dampen inflammation beyond the RAS, by using the cytokine interleukin (IL)-1β to activate human VSMC. Finally, we studied the contribution of Mas receptors subtypes to the actions of Ang-(1-7) and partly dissected the pro-inflammatory intracellular pathways modulated by Ang-(1-7) in human VSMC.

## Materials and Methods

### Materials

Culture plastic ware was from TPP (Trasadingen, Switzerland). Dulbecco’s Modified Eagle’s Medium (DMEM), fetal calf serum (FCS), and trypsin-EDTA were from Biological industries (Beit-Hamek, Israel). IL-1β was from Peprotech (London, UK), while the Mas antagonists A779 and D-Pro^7^-Ang-(1-7) were purchased Bachem (Bubendorf, Switzerland) and Biosyntan (Berlin, Germany), respectively. Anakinra was purchased from Swedish Orphan Biovitrum AB (Stockholm, Sweden). Ang-(1-7), Ang II, losartan, apocynin, and pyrrolidine dithiocarbamate (PDTC) were purchased from Sigma (St. Louis, MO, USA).

### Cell Culture

Primary cultures of human aortic smooth muscle cells (HASMC) were obtained by enzymatic dissociation from aortic fragments of five organ donors free of vascular disease (two men and three women, age 35 ± 5.6 years), conforming to the principles outlined in the Declaration of Helsinki and according to Spanish legal regulations, as previously described (Romacho et al., 2009). The study was approved by the Ethics Committee of Hospital Universitario de Getafe (CEIC 14/17). HASMC from the different donors were pooled and routinely cultured in DMEM supplemented with 10% FCS and antibiotics (Romacho et al., 2009). For experiments, HASMC were deprived of serum for 24 h prior to the addition of different test compounds in serum-free medium supplemented with 0.1% BSA. Cultures between the third and the tenth passages were used.

### Western Blot

The levels of iNOS were determined by Western blot using a polyclonal antibody (Transduction Laboratories, Lexington, KY, USA; dilution 1/1,000), followed by an appropriate secondary peroxidase-conjugated antibody (Chemicon, Temecula, CA, USA; dilution 1/10,000), as previously described (Romacho et al., 2009). Inmunoreactive bands were visualized by enhanced chemiluminescence detection (Millipore, Billerica, MA, USA), and quantified using ImageJ free software. For every experiment, iNOS levels were normalized to α-tubulin using a primary polyclonal antibody from Sigma (dilution 1/10,000).

### Determination of Nitric Oxide Release

Nitric oxide (NO) was indirectly quantified in HASMC supernatants using the Griess Method, as previously described (Lafuente et al., 2008). For results normalization, protein content was determined by the Bradford assay.

### Nuclear Extracts and Electromobility Shift Assay

Nuclear extracts were obtained as described before (Romacho et al., 2009). For electromobility shift assay (EMSA), a commercial oligonucleotide (Promega, Madison, WI, USA) encoding the NF-κB consensus sequence (5′-AGTTGAGGGGACTTTCCCAGGC-3′) was 5′-end labeled using [γ-^32^P]ATP and T4 polynucleotide kinase (Promega, Madison, WI, USA). For binding reactions, nuclear extracts (5 μg) were incubated on ice for 15 min in a reaction buffer [40 mM HEPES (pH 7.0), 140 mmol/L NaCl, 5 mM dithiothreitol, 10 μg/mL BSA, 0.01% Nonidet P-40, 4% Ficoll, and 0.05 μg/mL poly(dI-dC).poly(dI-dC)]. After addition of the labeled oligonucleotide (∼50,000 cpm) the reaction mix was further incubated for 20 min at room temperature. For competition experiments a 100-fold excess of unlabeled doubled-stranded oligonucleotide was added to the binding reaction. DNA-protein complexes were resolved on 4% non-denaturing polyacrylamide gels in 0.5x TBE (45 mmol/L Tris-borate, 1 mM EDTA, pH 8.0) at 4°C. Gels were dried and exposed to autoradiography at -80°C. The intensity of the resulting bands was quantified by densitometry, as previously described (Romacho et al., 2009), using Image J free software. The DNA binding activity of NF-κB was abolished by PDTC (100 μM).

### NADPH Oxidase Activity Assay

NADPH oxidase was measured by lucigenin-derived chemiluminescence, as described previously (Vallejo et al., 2014). Briefly, HASMC were washed in ice-cold phosphate-buffered saline, scraped and centrifuged. The resulting cell pellet was homogenized in lysis buffer (pH 7.0) containing 50 mM KH_2_PO_4_, 1 mM EGTA, and 150 mM sucrose. For every sample, the protein content was determined by the bicinchoninic acid method. Cell extracts (5 μg protein) were incubated in phosphate-buffered saline containing 5 μM lucigenin and 100 μM NADPH and luminiscence was then measured every 10 s for 5 min in a tube luminometer (Optocomp, MGM Instruments, Hamden, CT, USA). The enzymatic activity, which was sensitive to the blockade by apocynin (30 μM), was expressed as relative light units (RLUs)/μg of protein/min.

### Statistical Analysis

Results are expressed as mean ± SEM. Statistical analysis was performed using one-way ANOVA followed by Bonferroni *post hoc* test. *n* denotes the number of independent experiments. A *p-*value ≤0.05 was considered statistically significant.

## Results

### Ang-(1-7) Inhibits the Induction of iNOS by Ang II and IL-1β

**FIGURE 1:**
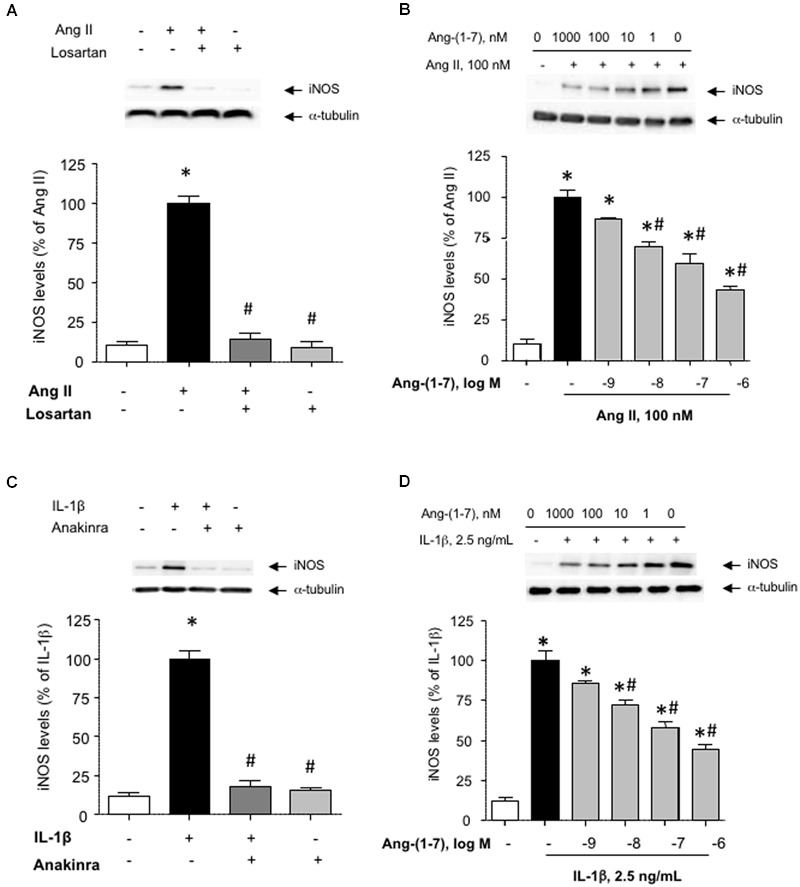
**Ang-(1-7) inhibits inducible nitric oxide synthase (iNOS) induction elicited by Ang II or IL-1β in human aortic smooth muscle cells (HASMC). (A)** HASMC were stimulated with Ang II (100 nM) alone or in the presence of the AT1 receptor antagonist losartan (1 μM) for 18 h, after which iNOS levels were determined by Western blot and normalized to α-tubulin. **(B)** In another set of experiments, the impact of increasing concentrations of Ang-(1-7) (1 nM–1 μM) on iNOS induction by Ang II was tested. **(C)** The pro-inflammatory cytokine IL-1β (2.5 ng/mL) promoted iNOS induction, which was prevented by the IL1 receptor blocker anakinra (1 μg/mL). **(D)** As for Ang II, Ang-(1-7) prevented the induction of iNOS by IL-1β in a concentration-dependent manner. Results are expressed as mean ± SEM; *n* = 5. Representative blots are shown in the corresponding panels. ^∗^*p* < 0.05 vs. untreated cultures, ^#^*p* < 0.05 vs. Ang II- or IL-1β-treated cultures, respectively.

The induction of iNOS, and the subsequent generation of NO is a major feature of the response of VSMC activated by pro-inflammatory stimuli (Chistiakov et al., 2015). **Figure 1A** shows that exposing HASMC to Ang II (100 nM) for 18 h resulted in a marked enhancement of iNOS levels, as compared with untreated cultures. This effect was abolished by the AT1 antagonist losartan (1 μM), which had no effect on iNOS levels by itself (**Figure 1A**). The induction of iNOS by Ang II was inhibited by Ang-(1-7) (1 nM–1 μM) in a concentration-dependent manner (**Figure 1B**).

While Ang-(1-7) has been gradually acknowledged as a physiological antagonist of Ang II, very little is known about the capacity of Ang-(1-7) to modulate the pro-inflammatory responses elicited by RAS-independent agonists. **Figure 1C** shows that the pro-inflammatory cytokine IL-1β (2.5 ng/mL) promoted the induction of iNOS in HASMC, which was blocked in the presence of the IL1 receptor antagonist anakinra (1 μg/mL). As for Ang II, Ang-(1-7) was capable to attenuate the induction of iNOS elicited by IL-1β (**Figure 1D**). For subsequent experiments, Ang-(1-7) was used at a fixed concentration of 100 nM.

### The Mas Antagonists A779 and D-Pro-Ang-(1-7) Prevent the Attenuation of iNOS Induction by Ang-(1-7)

**FIGURE 2:**
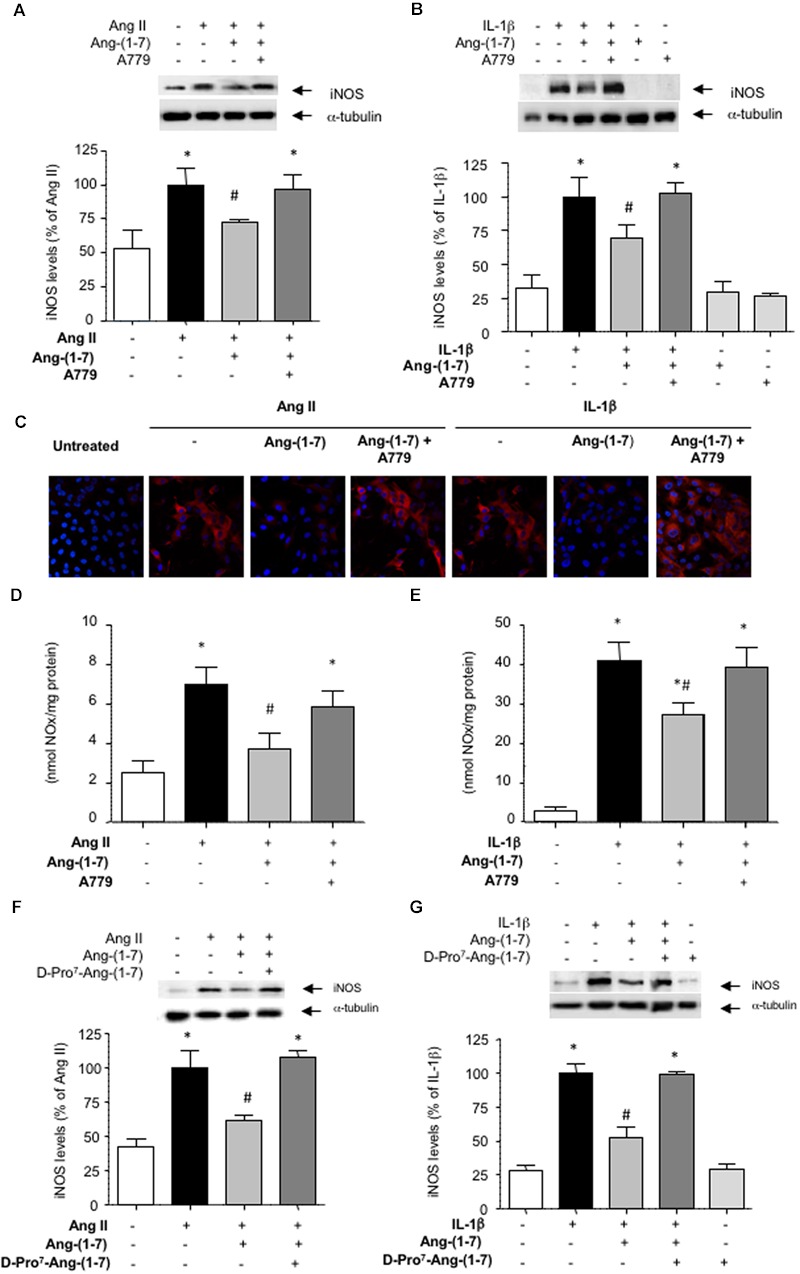
**The inhibition of iNOS induction mediated by Ang-(1-7) is dependent on Mas receptors.** After exposing HASMC for 18 h to **(A)** Ang II (100 nM) or **(B)** IL-1β (2.5 ng/mL) with or without 100 nM Ang-(1-7), iNOS levels were determined by Western blot. In some experiments, cells were pre-incubated with the Mas receptor antagonist A779 (1 μM). **(C)** iNOS levels (red) were also determined by indirect immunofluorescence in response to the different stimuli. Nuclei were counterstained with DAPI (blue) (400×). In another set of experiments, nitric oxide (NO) production was determined in the supernatants of HASMC exposed for 18 h to Ang II **(D)** or IL-1β **(E)**, with or without Ang-(1-7) and A779. The Mas receptor antagonist D-Pro^7^-Ang-(1-7) (1 μM) was also capable to block the inhibitory effect of Ang-(1-7) on iNOS induction by Ang II **(F)** or IL-1β **(G)**. Results are expressed as mean ± SEM; *n* = 5. Representative blots are shown in the corresponding panels. ^∗^*p* < 0.05 vs untreated cultures, ^#^*p* < 0.05 vs Ang II- or IL-1β-treated cultures, respectively.

Ang-(1-7) has been described as a ligand for the G-protein-coupled receptor Mas (Kostenis et al., 2005). In HASMC, the Mas receptor antagonist D-Ala^7^-Ang-(1-7) (A779; 1 μM) prevented the capacity of Ang-(1-7) to attenuate the induction of iNOS elicited by both Ang II and IL-1β, as shown by Western blot (**Figures 2A,B**) and by indirect immunofluorescence (**Figure 2C**). Accordingly, A779 also blocked the reduction of NO release elicited by Ang-(1-7) in cells stimulated with Ang II or IL-1β (**Figures 2D,E**). In the absence of Ang II or IL-1β, neither Ang-(1-7) nor A779 did modify by themselves iNOS levels (**Figure 2B**) or NO release (3.12 ± 0.93 and 2.98 ± 1.06 nmol/mg protein, respectively; *n* = 3–5).

The existence of several Mas receptor subtypes in the vasculature has been previously suggested based on the differential capacity of two Mas receptors blockers A779 and D-Pro^7^-Ang-(1-7) to fully counteract some biological actions of Ang-(1-7), such as vasodilation (Silva et al., 2007). In HASMC cultures, however, D-Pro^7^-Ang-(1-7) also blocked the inhibitory effects of Ang-(1-7) on iNOS induction (**Figures 2F,G**). D-Pro^7^-Ang-(1-7) alone did not modify iNOS levels (**Figure 2G**).

### Ang-(1-7) Prevents NF-κB Activation

**FIGURE 3:**
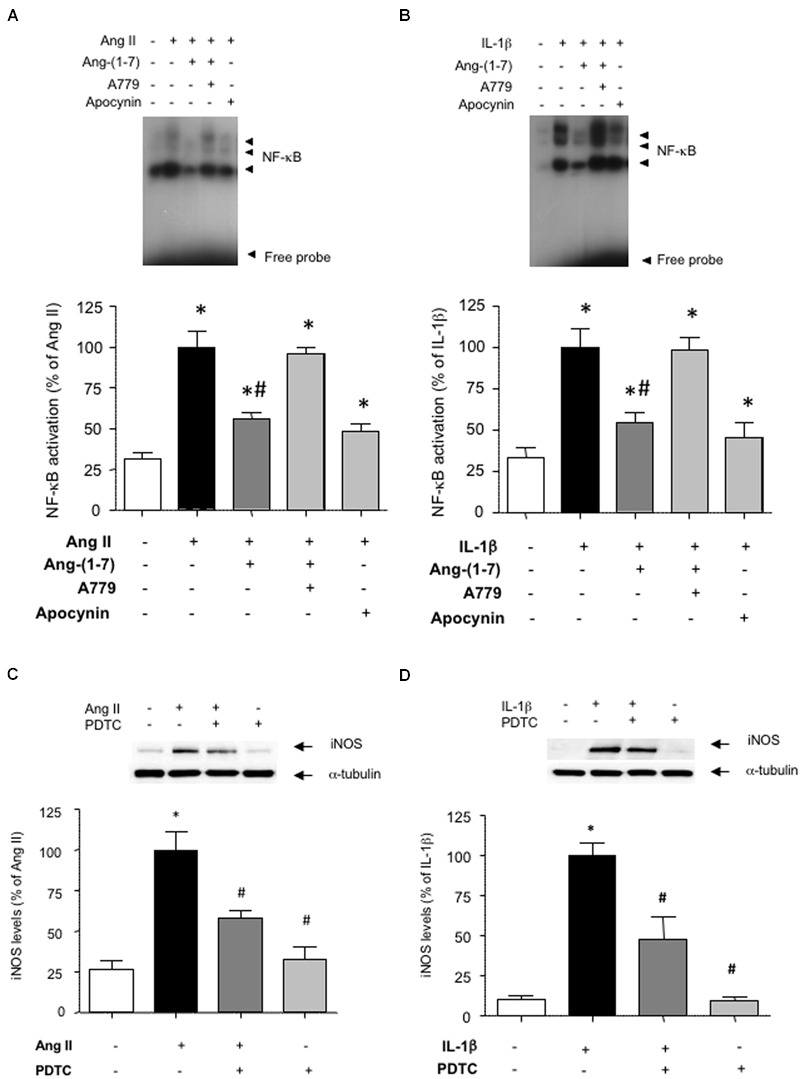
**Ang-(1-7) reduces NF-κB activation via Mas receptors in HASMC.** The activation of NF-κB induced after 18 h incubation with **(A)** Ang II (100 nM) or **(B)** IL-1β (2.5 ng/mL) was blunted by the antioxidant NADPH oxidase inhibitor apocynin (30 μM) and significantly reduced by Ang-(1-7) (100 nM), as determined by EMSA. The effect of Ang-(1-7) was prevented by the Mas receptor antagonist A779 (1 μM). The induction of iNOS by Ang II **(C)** or IL-1β **(D)** was abolished by the NF-κB inhibitor pyrrolidine dithiocarbamate (PDTC) (100 μM). Representative blot or EMSA images are shown in the corresponding panels. Results are expressed as mean ± SEM; *n* = 4. ^∗^*p* < 0.05 vs untreated cultures, ^#^*p* < 0.05 vs Ang II- or IL-1β-treated cultures, respectively.

To gain insight into the signaling pathways influenced by Ang-(1-7), we next focused on NF-κB, as a pivotal transcription factor regulating the expression of inflammation-related proteins, including iNOS (Brasier, 2010). Both Ang II and IL-1β promoted the activation of NF-κB (**Figures 3A,B**). This step that was necessary for the downstream induction of iNOS, since the NF-κB inhibitor PDTC abolished iNOS induction (**Figures 3C,D**). Ang-(1-7) was capable to attenuate NF-κB activation, and this effect was blocked by A779 (**Figures 3A,B**).

### Ang-(1-7) Inhibits the Stimulation of NADPH Oxidase Activity

**FIGURE 4:**
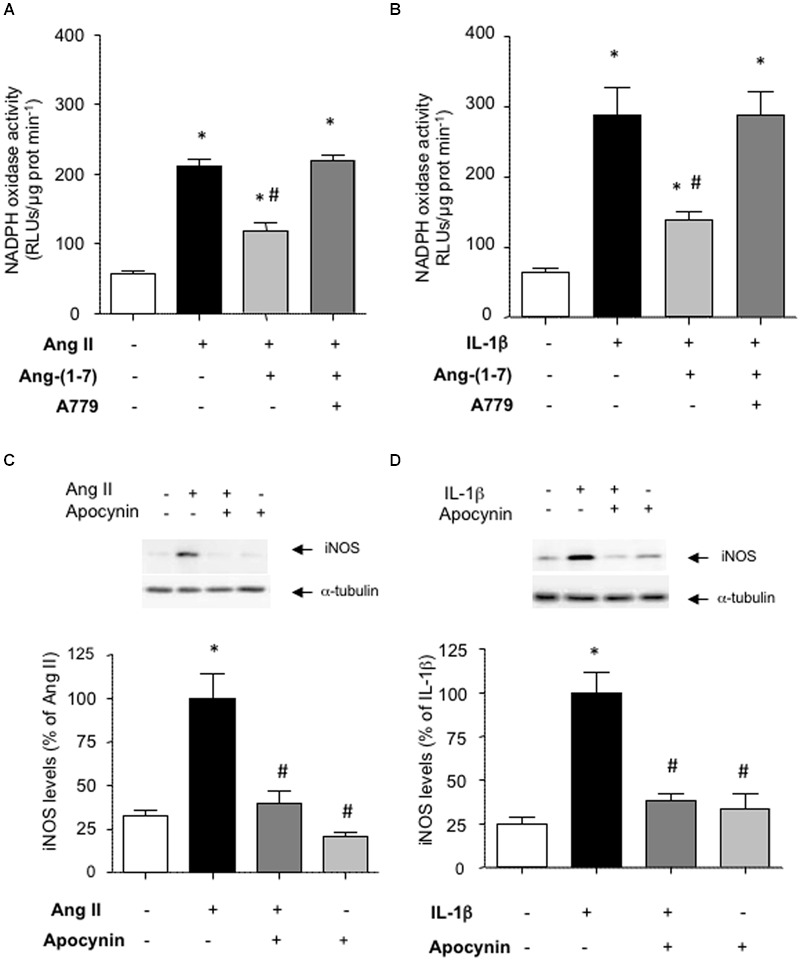
**Ang-(1-7) reduces NADPH oxidase activity via Mas receptors.** In HASMC, Ang-(1-7) (100 nM) reduced the stimulation of NADPH oxidase observed after 18 h incubation with **(A)** Ang II (100 nM) or **(B)** IL-1β (2.5 ng/mL), and this effect was blocked by the Mas receptor antagonist A779 (1 μM). The inhibition of NADPH oxidase by the antioxidant inhibitor apocynin (30 μM) prevented the induction of iNOS by Ang II **(C)** or IL-1β **(D)**. Representative blots are shown in the corresponding panels. The NADPH oxidase activity is given as relative light units (RLUs)/μg protein/min. Results are expressed as mean ± SEM; *n* = 4. ^∗^*p* < 0.05 vs untreated cultures, ^#^*p* < 0.05 vs Ang II- or IL-1β-treated cultures, respectively.

NF-κB is a redox-sensitive factor that can be regulated by different sources of ROS. Among these, NADPH oxidase is a major ROS-generating enzyme in vascular cells and its over-activation has been linked to vascular damage (Nguyen Dinh Cat et al., 2013). **Figures 4A,B** show that Ang-(1-7) was able to attenuate the stimulation of NADPH oxidase activity triggered by both Ang II and IL-1β in HASMC. The use of the antioxidant NADPH oxidase inhibitor apocynin revealed that NADPH oxidase activity was necessary for the consecutive downstream NF-κB activation (**Figures 3A,B**) and iNOS induction (**Figures 4C,D**) elicited by Ang II and IL-1β.

## Discussion

Since inflammation is at the basis of a wide array of cardiovascular diseases, the identification of pharmacological tools to prevent or attenuate vascular cell inflammation has gained growing attention over the last years (Natarajan and Cannon, 2010). In this study, we have identified Ang-(1-7) as a novel player in preventing the pro-inflammatory activation of human VSMC. More specifically, Ang-(1-7) attenuated the induction of iNOS, a NO-generating enzyme that is found over-activated in a series of vascular diseases running with low grade inflammation, such as atherosclerosis, hypertension or diabetic vasculopathy (Shimokawa and Tsutsui, 2010; Di Pietro et al., 2013; Oliveira-Paula et al., 2014).

Over a decade ago, Mas was identified as the main receptor mediating the biological actions of Ang-(1-7) (Santos et al., 2003). The *Mas* proto-oncogene encodes a 7 transmembrane protein exhibiting features of G-protein coupled receptors (Santos et al., 2003). In the vasculature, the presence of more than one subtype of Mas receptors was proposed by Silva et al. (2007) based on the capacity of D-Pro^7^-Ang-(1-7), but not of A779, to inhibit Ang-(1-7)-mediated vasodilation in rat aortas (Silva et al., 2007). More recently, an acute lung injury model has also pointed out the existence of more than one type of Mas receptors in the respiratory system (Klein et al., 2013). In contrast, we observed that D-Pro^7^-Ang-(1-7) and A779 were equivalent in blocking the effects of Ang-(1-7). This rather indicates that both antagonists target the same receptor in human VSMC and that a unique Mas subtype receptor thus mediates the anti-inflammatory effects of Ang-(1-7) in this cell type. Indeed, our findings are in line with other reports identifying a single Mas receptor, sensitive to both D-Pro^7^-Ang-(1-7) and A779 antagonism, mediating the vasorelaxant action of Ang-(1-7) in murine conductance and resistance arteries (Lemos et al., 2005; Peiró et al., 2013) as well as other non-vascular actions of the heptapeptide (Zhao et al., 2015).

Through its binding to Mas receptors, Ang-(1-7) attenuated the Ang II-stimulated induction of iNOS in human VSMC. Indeed, the ACE2/Ang-(1-7) axis is nowadays regarded as an endogenous counterpart for ACE/Ang II axis, and is thus considered to play a key role in regulating and limiting the actions of a central player in cardiovascular physiopathology such as Ang II. In the cardiovascular system, Ang-(1-7) has proven to counterbalance the vasoconstrictor, pro-oxidant, pro-fibrotic, and hypertrophic actions of Ang II (Grobe et al., 2007; McCollum et al., 2012). We now demonstrate that Ang-(1-7) may also protect against VSMC inflammation elicited by the main biologically active peptide of the RAS.

However, one of the major findings of the present study is that the Ang-(1-7)/Mas axis not only counteracts the action of Ang II, but is also acts as an anti-inflammatory compound beyond the RAS. Pro-inflammatory cytokines, including IL-1β, are key contributors to the low-grade inflammatory environment that characterizes a wide array of vascular diseases (Ridker, 2016). Indeed, high sensitivity C-reactive protein, used as a biomarker of cardiovascular risk, is now being considered as a downstream surrogate marker of upstream IL-1β activity (Ridker, 2016). From a pharmacological perspective, drugs targeted against IL-1β and its receptors, initially used to treat non-vascular inflammatory conditions, are now being assayed as vasculoprotective and anti-atherothrombotic tools (Ridker, 2016). In the present study, the stimulation of Ang-(1-7)/Mas axis revealed itself as an alternate strategy to minimize the impact of IL-1β on human VSMC pro-inflammatory activation. This is in line with other reports showing that in the context of neuroinflammation the pharmacological activation of the ACE2/Ang-(1-7) can reduce the inflammatory response of human retinal pigment epitelial cells to lipopolysaccharide (Tao et al., 2016). Furthermore, Ang-(1-7) may prevent the inflammation induced in astrocytes by physical stressors, such as radiation (Moore et al., 2013).

**FIGURE 5:**
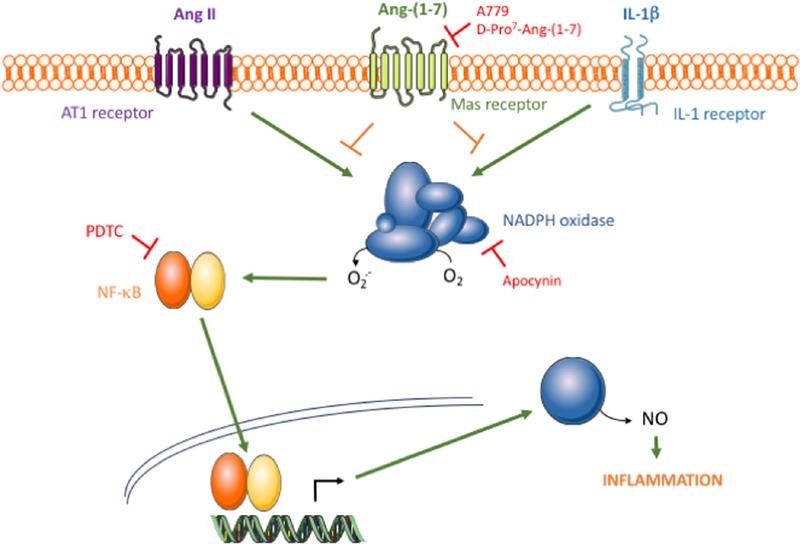
**Schematic diagram of the proposed action of the Ang-(1-7)/Mas axis on HAMSC pro-inflammatory signaling.** The binding of Ang II or IL-1β to their respective membrane receptors results in NADPH oxidase activation, which consecutively leads to NF-κB activation, iNOS induction and NO release. Through an effect mediated by Mas receptors, Ang-(1-7) promotes NADPH oxidase inhibition and thus attenuates the pro-inflammatory signaling triggered by Ang II, but also by other non-RAS inflammatory stimuli, such as IL-1β.

To further understand the mechanisms underlying the anti-inflammatory action of Ang-(1-7), the focus was set on common signaling pathways activated by both Ang II and IL-1β. The transcription factor NF-κB is a main redox-sensitive regulator of iNOS expression (Teng et al., 2002; Zahradka et al., 2002), and its over-activation by a wide array of stimuli has been associated to chronic inflammatory vascular diseases, such as atherosclerosis (Monaco et al., 2004). Upstream of NF-κB, Ang-(1-7) promoted the inhibition of NADPH oxidase and diminished ROS generation, which in turn led to a marked attenuation of the NF-κB/iNOS axis and thus of human VSMC activation (**Figure 5**). This is in line with other reports showing that Ang-(1-7) negatively regulates NADPH oxidase activation elicited by Ang II in endothelial cells (Sampaio et al., 2007; Xiao et al., 2015) or other non-vascular cell types, such as skeletal muscle or kidney tubular cells, among other (Kim et al., 2012; Morales et al., 2014). The activation of the IP3/Akt pathway, one of most relevant Mas-related signaling events acknowledged to date (Bader et al., 2014), might be on the basis of the protective effect of Ang-(1-7) against NOX-derived ROS formation and apoptosis in cerebral endothelial cells (Xiao et al., 2015). In embryonic pancreatic explants, however, Ang-(1-7) rather seems to activate NADPH oxidase (Wang et al., 2015), which indicates that the precise mechanisms linking Mas activation and NADPH oxidase activity may be complex and still remain to be explored more in depth. Overall, our findings show that the inhibition of NADPH oxidase activity appears as a pivotal common intracellular target of Ang-(1-7) in protecting against inflammation triggered by stimuli of different nature. This mechanism might indeed be on the basis of the beneficial action of the heptapeptide on experimental atherosclerosis plaque formation (Yang et al., 2015). Whether Ang-(1-7) can modulate other ROS sources and total redox balance remains to be further explored.

Over the last years, pharmacological research has been undertaken to identify valuable tools to regulate the ACE2/Ang-(1-7)/Mas axis, with particular focus on Ang-(1-7) mimetics and ACE2 activity modulators (Fraga-Silva et al., 2013; Simões e Silva et al., 2013). This study supports that activating or mimicking the ACE2/Ang-(1-7)/Mas axis may represent a valuable pharmacological opportunity to attenuate the pro-inflammatory environment that promotes and sustains the development of vascular diseases.

## Author Contributions

CP and CS-F designed the experiments. LV, EC, AR and SV generated experimental data. CP, CS-F, LV, EC, AS-L, AR, and MR-G analyzed raw data and performed statistical analysis. LV, AH-L, AR and EC drew the figures. CP and CS-F wrote the manuscript. LV, SV, TR and RC contributed in discussion and reviewed the manuscript.

## Conflict of Interest Statement

The authors declare that the research was conducted in the absence of any commercial or financial relationships that could be construed as a potential conflict of interest.
